# Immune‐Related Cystitis Following Immune Checkpoint Inhibitor Therapy: A Report of Two Cases

**DOI:** 10.1155/carm/7432835

**Published:** 2026-09-01

**Authors:** Chuanren Zhao, Yang Ge, Xiaojing Zhang

**Affiliations:** ^1^ Department of Cancer Center, Beijing Ditan Hospital, Capital Medical University, Beijing 100015, China, ccmu.edu.cn; ^2^ Oncology Department, Beijing Chaoyang Hospital, Capital Medical University, Beijing 100020, China, ccmu.edu.cn

**Keywords:** cystitis, immune checkpoint inhibitors, immune-related adverse events, PD-1 inhibitors

## Abstract

In recent years, immune checkpoint inhibitors (ICIs) have transformed the treatment landscape for a variety of cancers, yet they are associated with immune‐related adverse events (irAEs). Although irAEs commonly affect the skin, gastrointestinal tract, lungs, and endocrine glands, immune‐related cystitis and ureteritis are extremely rare and often overlooked. We report on two patients who developed immune‐mediated ureteritis and cystitis following treatment with PD‐1 inhibitors (tislelizumab and toripalimab). Both patients exhibited urinary frequency, urgency, dysuria, and hematuria without evidence of infection and were unresponsive to empirical antibiotics. Imaging results, combined with clinical symptoms, confirmed the diagnosis of urinary tract irAEs. Symptoms improved markedly following systemic glucocorticoid therapy. Through case analysis and a targeted literature review, we have summarized diagnostic indicators, therapeutic principles, and steroid tapering strategies for managing ICI‐associated urinary irAEs. We emphasize the importance of early recognition and standardized management to prevent permanent damage and offer practical guidance for clinicians dealing with these uncommon but clinically significant toxicities.

## 1. Introduction

Immune checkpoint inhibitors (ICIs) have become a landmark breakthrough in cancer treatment, exerting antitumor effects by blocking inhibitory immune pathways and restoring T‐cell–mediated tumor clearance [1]. These agents have achieved durable responses and survival benefits across numerous solid tumors, including gastric cancer, esophageal cancer, and lung cancer [2, 3]. However, the widespread activation of the immune system also leads to a spectrum of immune‐related adverse events (irAEs) that can affect nearly any organ system [4]. The most frequently affected organs include the skin, gastrointestinal tract, lungs, endocrine glands, and musculoskeletal system [5]. In comparison, immune‐related cystitis and ureteritis are extremely rare irAEs, with low reported incidence and a high risk of misdiagnosis as a urinary tract infection (UTI) [6, 7]. Patients typically present with urinary frequency, urgency, dysuria, and hematuria, accompanied by inflammatory changes in the bladder and ureteral walls on imaging, while urine cultures remain negative and symptoms do not respond to antibiotics [8]. These features create substantial diagnostic and therapeutic challenges in clinical practice.

This case report is clinically significant because it describes two well‐documented cases of immune‐related cystitis and ureteritis induced by programmed death‐1 (PD‐1) inhibitors (tislelizumab and toripalimab) in patients with advanced gastric and esophageal cancer. Both cases illustrate the typical clinical presentation, imaging characteristics, poor response to antibiotics, and favorable outcome after glucocorticoid therapy, providing a practical and reproducible diagnostic framework for this rare irAE. In addition, the report addresses a common and unresolved clinical problem: symptom recurrence during steroid tapering, which is often underreported but clinically impactful.

The novelty of this report is reflected in three major aspects:

1. Concomitant irAEs: To our knowledge, this is one of the first reports to describe a patient who developed both immune‐related pneumonitis and cystitis sequentially after toripalimab, suggesting that the occurrence of one irAE should raise vigilance for multiorgan immune toxicity.
2. Biomarker insight: We provide preliminary evidence that persistent elevation of interleukin‐17A (IL‐17A) may indicate subclinical inflammation and predict flare‐up during steroid tapering, suggesting a potential objective biomarker for safer and more individualized tapering strategies.
3. Practical guidance: By combining clinical experience, imaging findings, steroid dosing, and relapse management, this study provides actionable evidence for early recognition, intervention, and long‐term monitoring of ICI‐associated urinary tract irAEs.

Early and accurate diagnosis remains essential to avoid unnecessary antibiotic use, prevent permanent urinary tract damage, and ensure continued access to effective immunotherapy. This case series aims to increase clinicians’ awareness of ICI‐related cystitis and ureteritis, improve diagnostic accuracy, and optimize clinical management for these rare but clinically important toxicities.

## 2. Case Presentation

### 2.1. Case 1

**FIGURE 1:**
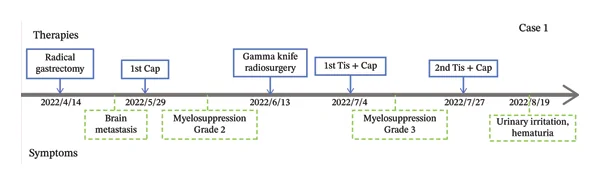
Clinical course of the patient in Case 1. Tis: tislelizumab; Cap: capecitabine.

The patient was a 58‐year‐old man, diagnosed in April 2022 with ulcerative, infiltrative, poorly differentiated adenocarcinoma of the stomach. The tumor included components of poorly cohesive carcinoma (with signet‐ring cells) and tubular adenocarcinoma. According to the Lauren classification, it was of the mixed type. The tumor had infiltrated the full thickness of the gastric wall, reaching the serosa and involving the pancreatic parenchyma. Metastatic carcinoma was detected in 15 of the 50 dissected lymph nodes. The pathological staging was pT4bN3. Immunohistochemistry revealed HER‐2 to be negative, while MLH1, MSH2, MSH6, and PMS2 were positive. EBER was negative upon in situ hybridization. Molecular testing indicated HER2 wild‐type status, microsatellite stability (MSS), and PD‐L1 expression with a tumor proportion score (TPS) of less than 1% and a combined positive score (CPS) of 0. In May 2022, the patient was admitted to our hospital and diagnosed with Stage IV poorly differentiated gastric adenocarcinoma (T4bN3aM1) with perigastric and retroperitoneal lymph node metastases. Following surgery, the patient received one cycle of single‐agent capecitabine starting on May 29, 2022. However, a subsequent brain MRI revealed multiple brain metastases, for which gamma knife radiosurgery was performed. The treatment regimen was then switched to two cycles of tislelizumab combined with capecitabine (starting on July 4th, after the first tislelizumab + capecitabine cycle on July 27th [Day 24]). The first day of using immunotherapy is marked as Day 1. During treatment, the patient experienced Grade 2–3 bone marrow suppression, manifesting as neutropenia. He had no personal history of hypertension, diabetes, kidney disease, or relevant family history of hereditary disease (Figure 1.).

**FIGURE 2:**
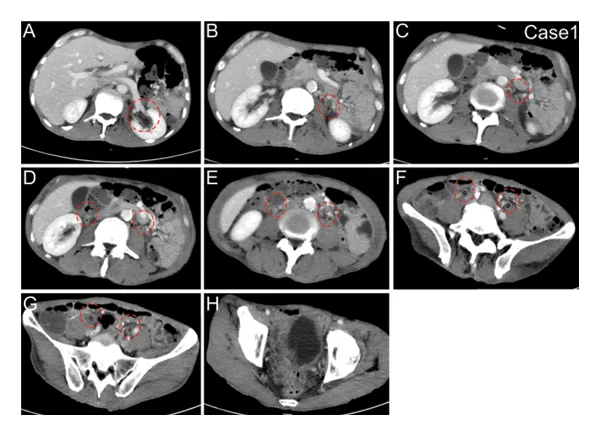
Case 1 patient’s CT scan revealed the inflammation of bilateral ureteral and bladder wall. The abdominal CT revealed mild bilateral hydronephrosis with mural thickening and enhancement of both ureters and the bladder, findings consistent with inflammation.

**FIGURE 3:**
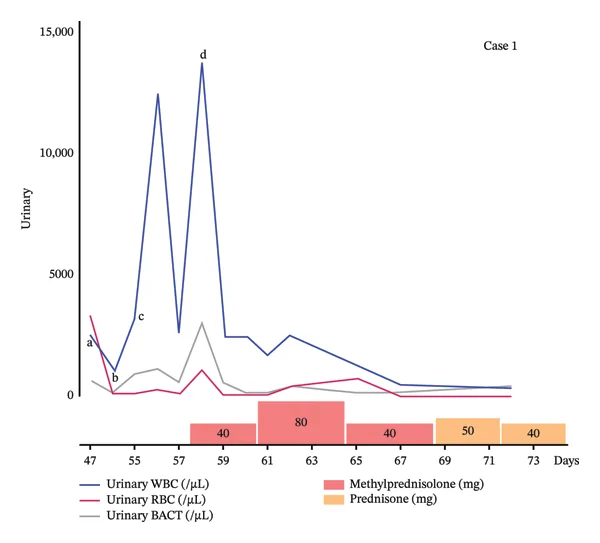
Number of days after tislelizumab dose in Case 1. (a = Day 47): Urinary urgency, dysuria with gross hematuria; levofloxacin was used. (b = Day 51): Levofloxacin was used. (c = Day 55): Disease progression and fever led to the switch from levofloxacin to cefoperazone–sulbactam. (d = Day 58): Low back pain, immune‐related cystitis was diagnosed.

On August 19, 2022 (Day 47), the patient presented at a urology outpatient clinic with sudden‐onset urinary urgency, dysuria, and gross hematuria without an identifiable precipitating factor. Urinalysis indicated a UTI, and empirical treatment with levofloxacin 0.5 g/day was initiated, with minimal improvement. The patient was admitted on August 23 (Day 51) for further treatment, including intravenous levofloxacin. Admission urinalysis revealed WBC 2518.9/μL, RBC 3249.8/μL, bacterial count 644/μL, and protein 300 mg/dL. By August 27 (Day 55), symptoms had worsened, accompanied by a fever peaking at 38.7°C. Laboratory tests showed procalcitonin at 0.41 ng/mL; a repeat urinalysis indicated WBC 12135.3/μL, RBC 280.8/μL, bacteria 1120.7/μL, and protein 100 mg/dL; a complete blood count showed WBC 4.25 × 10^9^/L, neutrophils 1.94 × 10^9^/L, RBC 2.95 × 10^9^/L, and platelets 199 × 10^9^/L. Antibiotic therapy was escalated to cefoperazone–sulbactam 3 g every 8 h, and urine cultures were obtained. On August 29 (Day 57), The patient’s body temperature returned to normal; however, on August 30 (Day 58), the patient experienced worsening flank pain with persistent lower urinary tract symptoms. Urinalysis now showed WBC 13367.0/μL, RBC 1047.4/μL, bacteria 2924.2/μL, and protein 100 mg/dL; fungal and bacterial smears/cultures and acid‐fast stains remained negative. Contrast‐enhanced abdominal computed tomography (CT) (Figure 2) revealed mild bilateral hydronephrosis with mural thickening and enhancement of both ureters and the bladder, findings consistent with inflammation. Given the negative microbiology, imaging, and temporal association with ICI therapy, immune‐mediated ureteritis and cystitis were strongly suspected. From August 30 to September 9 (Day 58–Day 69), the patient received intravenous methylprednisolone sodium succinate with dose adjustments. Urinary frequency, urgency, and hematuria progressively improved. The patient was discharged on September 10, prescribed prednisone acetate 50 mg/day, tapered by 10 mg every 10 days to 30 mg and then by 5 mg weekly until discontinuation in December 2022. No recurrence of urinary symptoms was noted during follow‐up (Figure 3.).

### 2.2. Case 2

**FIGURE 4:**
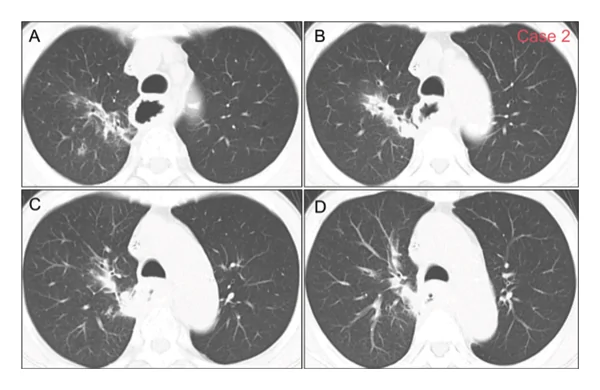
Case 2 patient’s chest CT showed immune‐related pneumonitis in the right lung. Chest CT scans (A) and (B) revealed Grade 2 immune‐related pneumonitis in the right lung following a cycle of toripalimab combined with chemotherapy. Scans (C) and (D) indicated improvement of the lesion compared to the previous scan, suggesting Grade 1 immune‐related pneumonitis after temporarily discontinuing toripalimab.

**FIGURE 5:**
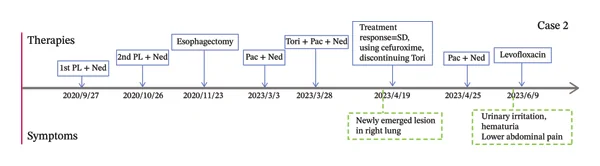
Clinical course of the patient in Case 2. PL: Paclitaxel liposome; Ned: nedaplatin; Tori: toripalimab; Pac: paclitaxel (albumin bound); SD: stable disease.

The patient was a 70‐year‐old male who initially presented in 2020 with progressive dysphagia. Gastroscopy revealed esophageal squamous cell carcinoma. He underwent two cycles of neoadjuvant chemotherapy in September and October 2020, consisting of liposomal paclitaxel 240 mg on Day 1 and nedaplatin 50 mg on Days 1–3. In November 2020, he underwent esophagectomy with esophagogastrostomy. Postoperative pathology confirmed moderately differentiated squamous cell carcinoma of the esophagus. In February 2023, routine follow‐up imaging revealed enlarged mediastinal lymph nodes, raising concern for recurrence and possible metastases. On March 2, 2023, the patient began first‐line systemic therapy for advanced disease (Cycle 1), which included the following: albumin‐bound paclitaxel 200 mg on Day 1 and 100 mg on Day 8 + nedaplatin 40 mg on Day 1 and 30 mg on Days 2–3, administered every 21 days (q21d). PD‐L1 immunohistochemistry on the previous surgical specimen showed a TPS of 30%. On March 28, 2023 (Day 1 of Cycle 2), the treatment regimen was modified to include toripalimab 240 mg on Day 1 while maintaining the chemotherapy components: Albumin‐bound paclitaxel 200 mg on Day 1 and 100 mg on Day 8 + nedaplatin 40 mg on Day 1, 30 mg on Days 2–3, q21d. A follow‐up evaluation on April 19, 2023, showed stable disease (SD). However, chest CT (Figure 4A,B) revealed a newly developed lesion in the right lung, which was considered to represent Grade 2 immune‐related pneumonitis. As a precaution, toripalimab was temporarily discontinued. On April 25, 2023, the patient proceeded with Cycle 3 of chemotherapy alone: albumin‐bound paclitaxel 200 mg on Day 1, 100 mg on Day 8 + nedaplatin 40 mg on Day 1, 30 mg on Days 2–3, q21d. On May 29, 2023 (Day 63), follow‐up chest CT (Figure 4C,D) indicated improvement of the lesion compared to the previous scan, suggesting Grade 1 immune‐related pneumonitis; toripalimab (Cycle 4) 240 mg was reintroduced in combination with chemotherapy: toripalimab 240 mg on Day 1 + albumin‐bound paclitaxel 200 mg on Day 1 + nedaplatin 40 mg on Days 1–2, q21d (Figure 5).

On June 9, 2023, the patient experienced lower abdominal discomfort, urinary frequency, urgency, dysuria, and visible gross hematuria without any apparent causative factors. Initially assessed in the urology outpatient clinic, a UTI was suspected, and empiric levofloxacin was prescribed. However, as symptoms persisted, the patient was readmitted for further evaluation.

**FIGURE 6:**
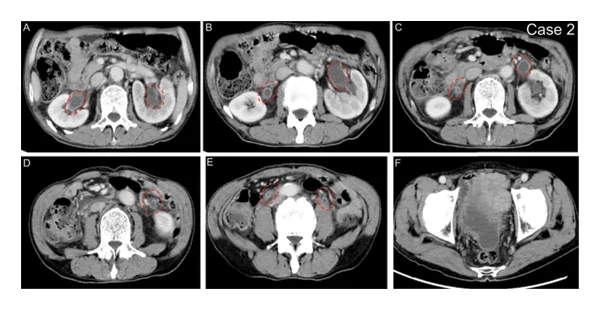
Case 2 patient’s CT scan revealed the inflammation of bilateral ureteral and bladder wall. The abdominal CT scan revealed mild dilatation of the bilateral renal pelves and ureters, accompanied by thickening of the ureteral walls. The bladder wall appears thickened with an irregular contour, suggesting an inflammatory process.

On June 16, 2023 (Day 81), the urinalysis indicated the following: WBC: 1365.0/μL, RBC: 174.9/μL, bacteria: 193.8/μL, urine WBC (dipstick): ++ (75/μL), urine RBC (dipstick): +++ (250/μL), and proteinuria: +++ (300 mg/dL). The complete blood count revealed the following: WBC: 7.75 × 10^9^/L, neutrophils: 85.8%, absolute neutrophils: 6.65 × 10^9^/L, lymphocytes: 0.78 × 10^9^/L, and C‐reactive protein (CRP): 13.220 mg/L. In line with Case 1, the urine culture was negative. Empirical treatment with ceftriaxone plus levofloxacin did not result in any improvement. An abdominal CT scan (Figure 6) showed mild bilateral hydronephrosis and ureteral wall thickening, indicating inflammation. After consultation with an infectious disease specialist, bacterial cystitis was deemed unlikely, and immune‐related cystitis was considered the most probable diagnosis.

**FIGURE 7:**
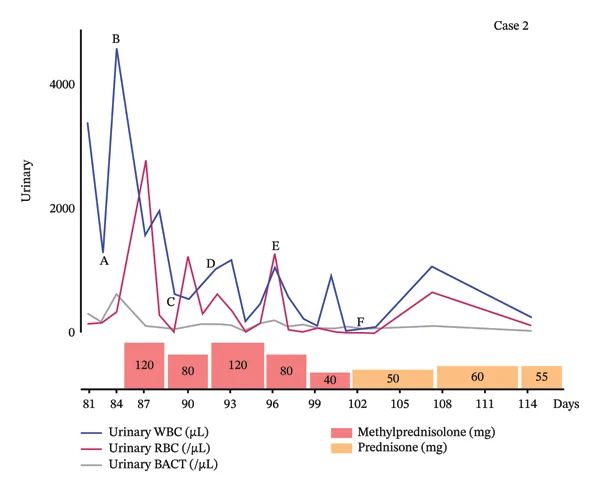
Number of days after toripalimab dose in Case 2. (A = Day 83): Frequent and painful urination, along with right lower abdominal pain. (B = Day 84): Right lower abdominal pain and urinary frequency have improved. (C = Day 89): Terminal hematuria, urinary frequency, and dysuria. (D = Day 92): Low back pain and cloudy urine, IL‐17A: 45.65 pg/mL. (E = Day 96): IL‐17A: 16.98 pg/mL. (F = Day 102): IL‐17A: 54.98 pg/mL.

On June 20, 2023 (Day 85), methylprednisolone was initiated at a dosage of 2 mg/kg/day. The patient’s urinary symptoms, which included frequency, urgency, dysuria, and flank pain, significantly improved following glucocorticoid therapy. However, as tapering began, his symptoms, particularly flank pain, recurred despite a downward trend in urinalysis markers (WBC, RBC, and bacteria). The steroid dose was then increased to 2 mg/kg/day (120 mg/day), which led to the recontrol of symptoms. Subsequent gradual tapering was reinitiated, and the patient was eventually discharged in an improved condition. Throughout the episode, the patient remained afebrile, with a maximum body temperature of 36.5°C (Figure 7).

## 3. Discussion

Tislelizumab and toripalimab are monoclonal antibodies targeting PD‐1, classified as ICIs. By blocking the PD‐1/PD‐L1 signaling pathway, they restore and enhance T‐cell–mediated antitumor activity. According to the Chinese Society of Clinical Oncology (CSCO) treatment guidelines [2, 3], both agents are approved for the treatment of various malignancies, including gastric cancer, esophageal cancer, non–small cell lung cancer, and hepatocellular carcinoma. IrAEs associated with ICI therapy can affect multiple organ systems, most commonly the skin, gastrointestinal tract, lungs, endocrine glands (including the thyroid, adrenal glands, and pituitary), and the musculoskeletal system [4, 5]. However, involvement of the bladder and urothelium has been rarely reported in the literature and is often under‐recognized in clinical practice.

We reported two cases of immune‐related cystitis and ureteritis following treatment with anti–PD‐1 monoclonal antibodies. In Case 1, the primary tumor was gastric cancer, treated with tislelizumab in combination with chemotherapy. In Case 2, the patient had esophageal squamous cell carcinoma and received toripalimab combined with chemotherapy. In both cases, patients developed urinary frequency, urgency, and dysuria without any identifiable infectious or mechanical triggers.

In Case 1, the patient presented with early‐onset fever, peaking at 38.7°C, which resolved following anti‐infective therapy. Conversely, the patient in Case 2 remained afebrile throughout the clinical course. Both patients exhibited lower urinary tract symptoms, including frequency and urgency, and urinalysis revealed elevated erythrocyte and leukocyte counts. However, repeated urine cultures were consistently negative, and empirical anti‐infective treatment yielded no clinical improvement. Notably, symptom resolution and a decrease in urinary red and white blood cell counts were observed only after the initiation of corticosteroid therapy. These findings suggest that the UTI‐like presentation may represent mild urothelial inflammation secondary to immune activation. Furthermore, neither patient exhibited hypotension, hypoxemia, or multiorgan dysfunction. Given that cytokine release syndrome (CRS) is defined as a systemic inflammatory response syndrome triggered by massive cytokine release (e.g., IL‐6, IL‐1, IFN‐γ, and TNF‐α) following hyperactivation of immune cells [9], the clinical presentation in our cases does not align with CRS characteristics. Therefore, CRS was excluded from our primary differential diagnosis.

Additionally, gross hematuria was observed in Case 2. Urinalysis in both patients revealed varying degrees of elevated RBC, WBC, and bacterial counts, yet repeated urine cultures and smears remained negative. Abdominal CT scans in both cases demonstrated diffuse thickening and enhancement of the ureteral and bladder walls, suggesting inflammatory changes. Symptoms did not improve with discontinuation of ICI therapy and empirical antibiotic treatment. A multidisciplinary team (MDT) consultation was convened, leading to the clinical suspicion of immune‐related cystitis in Case 1. The urology team recommended prompt cystoscopy and bladder biopsy should urinary symptoms fail to improve or deteriorate despite glucocorticoid therapy. Fortunately, the patient exhibited a marked symptomatic response to systemic glucocorticoid administration, corroborating the diagnosis of irAEs involving the urinary tract; consequently, cystoscopic evaluation was deferred. Given the striking clinical and therapeutic similarities between the two cases, an additional urological consultation was not pursued for Case 2 during the initial management phase.

The absence of cystoscopic evaluation represents a key limitation of this study. We emphasize that all patients with suspected immune‐related urinary adverse events should undergo standardized urological consultation, even in the presence of atypical clinical manifestations. This recommendation is intended to optimize diagnostic decision‐making and preclude missed diagnoses that necessitate endoscopic confirmation.

Based on the clinical manifestations and treatment courses of the two patients, combined with the recommendations of the Common Terminology Criteria for Adverse Events (CTCAE) Version 5.0 [10] and the guidelines for CSCO management of ICI‐related toxicity (CSCO guidelines) [11], the inflammatory grade in both patients was rated as Grade 3 (Tables 1 and 2). Cystoscopy combined with biopsy is the gold standard for the definitive diagnosis of immune‐associated cystitis and ureteritis in clinical practice. Regarding glucocorticoid dosing, the CSCO guidelines [11] (Table 3) recommend initiating methylprednisolone or prednisone at 1–2 mg/kg/day for patients experiencing Grade 3 genitourinary toxicities during ICI therapy. In Case 1, the patient had a height of 168 cm, weight of 43 kg, and a BMI of 15.2 kg/m^2^, indicating an underweight status. Based on the guideline‐recommended starting dose of 1 mg/kg/day, methylprednisolone was initiated at 40 mg/day (August 30–September 1, Days 58–60). The patient’s urinary frequency, urgency, and dysuria improved, and urinalysis indicated a decrease in both white and red blood cells. However, the patient developed worsening flank pain, suggesting suboptimal inflammatory control. The steroid dose was then increased to 80 mg/day (from September 2 to September 5, Days 61–64), resulting in a marked improvement in flank discomfort. The glucocorticoid was gradually tapered thereafter, and the patient was discharged on oral prednisone without recurrence of urinary symptoms. In Case 2, the patient measured 168 cm in height and weighed 58 kg (BMI = 20.5 kg/m^2^). Based on clinical experience from Case 1 and the patient’s better nutritional status, treatment was initiated at 2 mg/kg/day, with methylprednisolone 120 mg/day administered (from June 20 to June 23, Days 85–88). Urinary symptoms improved markedly. The dose was then tapered to 80 mg/day (from June 24 to June 26, Days 89–91), but the patient experienced a relapse of urinary frequency and urgency, along with flank pain. Urinalysis again showed elevated WBC and RBC counts. Consequently, the steroid dose was increased back to 120 mg/day (from June 27 to June 30, Days 92–95), which led to gradual symptom relief. The steroid was then tapered progressively, and the patient was discharged on oral glucocorticoids.

**TABLE 1:** CTCAE Version 5.0.

CTCAE term	Grade 1	Grade 2	Grade 3	Grade 4	Grade 5
Cystitis noninfective	Microscopic hematuria; minimal increase in frequency, urgency, dysuria, or nocturia; new onset of incontinence	Moderate hematuria; moderate increase in frequency, urgency, dysuria, nocturia, or incontinence; urinary catheter placement or bladder irrigation indicated; limiting instrumental ADL	Gross hematuria; transfusion, IV medications, or hospitalization indicated; elective invasive intervention indicated	Life‐threatening consequences; urgent invasive intervention indicated	Death

**TABLE 2:** CSCO management of immune checkpoint inhibitor–related bladder toxicity.

Grade 1	Grade 2	Grade 3	Grade 4
Asymptomatic or mild symptoms; clinical or diagnostic observation only	Moderate symptoms; interference with instrumental activities of daily living (IADL)	Severe symptoms; intravenous drug administration and hospitalization are required	Life‐threatening

**TABLE 3:** CSCO principles of toxicity grading and management.

Grade	Hospitalization level	Glucocorticoids	Other immunosuppressants	ICIs therapy
Grade 1	No hospitalization required	Not recommended	Not recommended	Continue use
Grade 2	No hospitalization required	Topical glucocorticoids or systemic glucocorticoids (oral prednisone, 0.5∼1 mg/(kg·d))	Not recommended	Hold use
Grade 3	Hospitalization required	Systemic glucocorticoid therapy: oral prednisone or intravenous methylprednisolone 1∼2 mg/(kg·d), followed by gradual tapering	For patients with no symptom relief after 2–5 days of glucocorticoid therapy, use may be considered under the guidance of a specialist physician	Discontinue use. Discuss whether to resume ICI therapy based on the patient’s risk–benefit ratio
Grade 4	Hospitalization required; consider admission to intensive care unit (ICU)	Systemic glucocorticoid therapy: intravenous methylprednisolone 1∼2 mg/(kg·d) for 3 consecutive days; if symptoms improve, gradually taper to 1 mg/(kg·d) for maintenance, then taper further and discontinue over 4–6 weeks	For patients with no symptom relief after 2–5 days of glucocorticoid therapy, use may be considered under the guidance of a specialist physician	Permanently discontinue

Based on the CSCO guidelines [11] recommendations and our institution’s clinical experience, we suggest that for patients diagnosed with immune‐related cystitis, initiating corticosteroid therapy at a dose of 2 mg/kg/day may facilitate more rapid symptom control. However, the optimal timing for steroid tapering remains uncertain. We reviewed the literature on ureteritis and cystitis associated with ICIs [6–8, 12–14] (Table 4). In one reported case [8], symptoms resolved after discontinuation of immunotherapy alone. In 10 additional cases [6, 8, 13, 14], symptom relief was achieved following corticosteroid administration. Notably, in 4 cases [7, 12], symptom recurrence occurred during steroid tapering, highlighting a potential risk of premature dose reduction. In Case 2, after four days of treatment with methylprednisolone (mPSL) at 2 mg/kg/day, laboratory tests indicated a downward trend in urinary WBC, RBC, and bacterial counts, and the patient’s urinary symptoms started to improve. Based on this initial response, we reduced the mPSL dose to 80 mg/day. However, during the tapering phase, urinalysis revealed a resurgence in WBCs and RBCs, and the patient experienced flank pain and irritative urinary symptoms once more. This observation suggests that improvement in urinary symptoms and urinalysis findings alone may be insufficient to determine the optimal timing for corticosteroid tapering. Additional clinical markers or standardized criteria are needed to guide the tapering process and prevent relapse.

**TABLE 4:** Summary of 9 cases of immune checkpoint inhibitor–related cystitis.

Case	Sex	Age	Carcinoma	ICIs	Pathology of urothelium	Treatment
1 [8]	Male	60	Squamous cell lung carcinoma	Nivolumab	Undone	Discontinuing nivolumab
2 [8]	Male	50	Squamous cell lung carcinoma	Nivolumab	Undone	PSL 60 mg/day (1 mg/kg/day)
3 [6]	Male	51	Small cell lung cancer	Nivolumab	CD3^+^ CD8^+^	mPSL 80 mg twice daily
4 [12]	Female	67	Breast cancer	Atezolizumab	CD8^+^, TIA‐1^+^	PSL 40 mg/day (1 mg/kg) ⟶ 30 mg/day ⟶ recurred ⟶ 60 mg/day
5 [13]	Male	53	Pulmonary adenocarcinoma	Sintilimab	Undone	mPSL 80 mg/day (1 mg/kg/day)
6 [14]	Male	61	Metastatic melanoma	Nivolumab	CD3^+^, CD20^+^, PD1^+^	PSL 0.5 mg/kg/day
7 [7]	Male	49	Esophageal carcinoma	Tislelizumab	CD3^+^, CD8^+^, CD20^+^, CD117^+^	mPSL 60 mg/day (1.5 mg/kg/day) ⟶ tapered gradually ⟶ recurred ⟶ 40 mg/day
8 [7]	Female	62	Gastric carcinoma	Sintilimab	Undone	mPSL 60 mg/day (1.7 mg/kg/day) ⟶ PSL 45 mg/day ⟶ recurred ⟶ PSL 30 mg/day
9 [7]	Male	49	Gastric carcinoma	Nivolumab	Undone	mPSL 60 mg/day (1.7 mg/kg/day) ⟶ recurred after assertion ⟶ mPSL 16 mg/day ⟶ mPSL 10 mg/day

*Note:* TIA‐1: T‐cell intracellular antigen 1; mPSL: methylprednisolone; PSL: prednisolone.

To explore whether adjunctive tools could assist in determining the optimal timing for corticosteroid tapering, we conducted cytokine profiling during the treatment course of Case 2 (refer to Table 5). IL‐1β is a potent proinflammatory cytokine that plays a critical role in host defense responses to infection and tissue injury. By binding to its receptor (IL‐1R), it activates downstream signaling pathways such as MAPK and NF‐κB, triggering broad inflammatory cascades. IL‐1β is involved in the pathogenesis of numerous diseases, including rheumatoid arthritis, autoimmune disorders, and the tumor microenvironment [15, 16]. IL‐17A, primarily produced by Th17 cells, acts synergistically with TNF‐α and IL‐1β to amplify inflammatory responses. It also promotes the production of other proinflammatory cytokines (e.g., IL‐6) and matrix‐degrading enzymes (e.g., MMPs) by a variety of cells, thereby contributing to tissue inflammation and damage [17]. In Case 2, on June 30, 2023, when the steroid dose had been tapered to 80 mg/day, we observed a marked reduction in most cytokines, with all returning to normal levels except for IL‐17A. However, three days later, on July 3, the patient experienced a clinical relapse, with worsening urinary symptoms and deteriorating urinalysis findings. After reescalation of corticosteroids, the patient’s condition improved again. Based on this observation, we hypothesize that persistent elevation of IL‐17A may be associated with ongoing subclinical inflammation and a risk of disease flare and thus may serve as a potential biomarker to guide steroid tapering decisions. However, this hypothesis requires further validation in a larger cohort.

**TABLE 5:** Cytokine detection in case 2.

Analyte name	Result pg/mL			Reference range pg/mL
	2023.6.26	2023.6.30	2023.7.6	
IL‐1β	56.79	0.96	66.09	0–2.54
IL‐17A	45.65	16.98	54.98	0–4.47
IL‐2	1.83	5.28	3.30	0–6.64
IL‐4	2.24	3.07	2.07	0–4.19
IL‐6	4.25	5.57	5.36	0–11.09
IL‐8	2.58	6.20	6.14	0–15.71
IL‐10	2.64	0.70	1.63	0–4.5
IL‐12P70	0.89	0.74	0.94	0–10.18
IL‐17F	0.73	1.09	1.10	0–4.66
IL‐22	1.26	0.82	2.15	0–3.64
IFN‐γ	7.26	2.99	4.54	0–4.43
TNF‐β	4.66	1.77	2.03	0–2.54

IrAEs are toxicities resulting from nonspecific activation of the immune system and can potentially involve almost any organ system. However, the precise mechanisms underlying irAEs have not yet been fully elucidated and may involve the activation of multiple inflammatory cell types, including Th17 cells and others [18]. IrAEs can occur at any stage of ICI therapy but most frequently develop within weeks to months after treatment initiation [19]. Based on previously reported cases of ICI‐associated cystitis, the onset of cystitis has ranged from 1 to 12 months after starting ICIs, corresponding to approximately treatment cycles 2–18 [6–8, 12–14].

In our report, both cases developed immune‐related ureteritis and cystitis after two cycles of anti–PD‐1 therapy. Notably, Case 1 did not exhibit any other systemic irAEs, such as cutaneous, endocrine, pulmonary, or gastrointestinal involvement. In contrast, Case 2 experienced suspected immune‐related pneumonitis following the first cycle of toripalimab combined with chemotherapy. Although symptoms partially improved with discontinuing toripalimab, pulmonary inflammation worsened after the second cycle and significantly improved only after corticosteroid administration. This indicates that Case 2 developed both immune‐related pneumonitis and cystitis, which, to our knowledge, represents the first such report in the literature. These findings highlight an important clinical implication: When one irAE is identified, clinicians should maintain a high index of suspicion for additional irAEs, enabling early diagnosis and timely intervention.

## Author Contributions

Writing–original draft preparation, Chuanren Zhao; writing–review and editing, Xiaojing Zhang and Yang Ge; clinical management, Chuanren Zhao, Xiaojing Zhang, and Yang Ge.

## Funding

No funding was received for this manuscript.

## Disclosure

All authors have read and agreed to the published version of the manuscript.

## Ethics Statement

Ethical review and approval were waived for this study because it involves case reports. Case reports typically involve the presentation of clinical observations without experimental interventions, and data are anonymized to protect patient confidentiality.

## Consent

Written informed consent has been obtained from the patients to publish this paper.

## Conflicts of Interest

The authors declare no conflicts of interest.

## Data Availability

Data are contained within the article.
